# The truth about metagenomics: quantifying and counteracting bias in 16S rRNA studies

**DOI:** 10.1186/s12866-015-0351-6

**Published:** 2015-03-21

**Authors:** J Paul Brooks, David J Edwards, Michael D Harwich, Maria C Rivera, Jennifer M Fettweis, Myrna G Serrano, Robert A Reris, Nihar U Sheth, Bernice Huang, Philippe Girerd, Vaginal Microbiome Consortium (additional members), Jerome F Strauss, Kimberly K Jefferson, Gregory A Buck

**Affiliations:** Department of Statistical Sciences and Operations Research, Virginia Commonwealth University, 23284-3083, Richmond, VA USA; Center for the Study of Biological Complexity, Virginia Commonwealth University, 23284, Richmond, VA USA; Department of Microbiology and Immunology, Virginia Commonwealth University, 23284, Richmond, VA USA; Department of Biology, Virginia Commonwealth University, 23284, Richmond, VA USA; Department of Obstetrics and Gynecology, Virginia Commonwealth University, 23284, Richmond, VA USA

**Keywords:** Assessments of microbial community structure via metagenomics, DNA extraction bias, PCR bias, Quality control, Next generation sequencing

## Abstract

**Background:**

Characterizing microbial communities via next-generation sequencing is subject to a number of pitfalls involving sample processing. The observed community composition can be a severe distortion of the quantities of bacteria actually present in the microbiome, hampering analysis and threatening the validity of conclusions from metagenomic studies. We introduce an experimental protocol using mock communities for quantifying and characterizing bias introduced in the sample processing pipeline. We used 80 bacterial mock communities comprised of prescribed proportions of cells from seven vaginally-relevant bacterial strains to assess the bias introduced in the sample processing pipeline. We created two additional sets of 80 mock communities by mixing prescribed quantities of DNA and PCR product to quantify the relative contribution to bias of (1) DNA extraction, (2) PCR amplification, and (3) sequencing and taxonomic classification for particular choices of protocols for each step. We developed models to predict the “true” composition of environmental samples based on the observed proportions, and applied them to a set of clinical vaginal samples from a single subject during four visits.

**Results:**

We observed that using different DNA extraction kits can produce dramatically different results but bias is introduced regardless of the choice of kit. We observed error rates from bias of over 85% in some samples, while technical variation was very low at less than 5% for most bacteria. The effects of DNA extraction and PCR amplification for our protocols were much larger than those due to sequencing and classification. The processing steps affected different bacteria in different ways, resulting in amplified and suppressed observed proportions of a community. When predictive models were applied to clinical samples from a subject, the predicted microbiome profiles were better reflections of the physiology and diagnosis of the subject at the visits than the observed community compositions.

**Conclusions:**

Bias in 16S studies due to DNA extraction and PCR amplification will continue to require attention despite further advances in sequencing technology. Analysis of mock communities can help assess bias and facilitate the interpretation of results from environmental samples.

**Electronic supplementary material:**

The online version of this article (doi:10.1186/s12866-015-0351-6) contains supplementary material, which is available to authorized users.

## Background

Next-generation sequencing technology (NGS) allows a much deeper characterization of the structure of microbial communities using metagenomic approaches. Metagenomic surveys often use a hypervariable region of the highly-conserved and universal 16S rRNA gene as a phylogenetic marker. Bias introduced in the processing steps of such surveys masks the true community composition so that there are large discrepancies in the proportion of gram negative bacteria observed using next generation sequencing, microscopy, and culture-based methods [1]. An objective of microbiome experiments is to characterize the community composition, including the relative quantities of species in sampled environments. An accurate depiction of microbial community composition via next generation sequencing requires a careful consideration of bias introduced during sample processing [2,3].

Many sources of bias have been identified in 16S rRNA studies using NGS including PCR amplification [4-11], DNA extraction protocol [5,12,13], sequencing artifacts [8,14-18], DNA copy number [19], sampling depth [7,20,21], and primer design [22-25]. Previous studies typically isolate one or two sources of bias, suggest experimental practices that mitigate the effects, and acknowledge that other sources of bias remain. Examples of recommendations for mitigating bias include performing triple DNA extraction [12], using multiple combinations of DNA extraction and PCR amplification protocols [5], and reducing the number of PCR cycles to avoid chimera formation [6].

Few studies have attempted to create models for neutralizing bias in environmental samples. One exception is a strategy proposed for counteracting the portion of bias due to differences in DNA copy number among bacteria [19]. The method applies a phylogenetic and ancestral state placement of sample sequences among a reference database of bacteria with known 16S copy numbers.

We perceive the need for three kinds of quality control in microbiome experiments. The first is the need to monitor batch effects of different sample processing runs. The use of identical mock (or even environmental) samples as positive controls and sequencing pure PCR product as a negative control can help to identify problems with batches and drift. The second type of quality control is based on the variation produced by the choice of sample processing protocols. The same sample processed at labs that use different protocols can produce different results. The Microbiome Quality Control project [3] is studying the effects of different choices in protocols and seeks to understand which choices contribute the most to variation. The third type of quality control is understanding the difference between observed and actual community compositions for particular choices of protocols for a lab. Depending on the environment of interest, labs will engineer their procedures so that they are sure to detect organisms of interest for the particular environment. Understanding the bias resulting from these choices of protocols is important because no matter what choice is made, bias will remain. The experiment reported here was designed to understand the magnitude and nature of bias introduced by a particular choice of protocols.

The methodology proposed here represents, to our knowledge, the first attempt to (1) create comprehensive models for predicting community composition in environmental samples based on observed proportions, (2) quantify the contribution of bias of different sample processing steps in 16S experiments, and (3) identify statistically significant relationships between bacterial signals. Additional distinguishing features of this study include the deep sequencing employed and the species-level taxonomic classification of reads.

This paper proposes a set of mixture experiments involving small “mock” communities, artificial microbial communities created by mixing known quantities of bacterial isolates, DNA clones, or PCR product. Mock communities are often used for ground-truthing and quantifying bias [4,9]. While mixture experiments occur most frequently in areas such as chemistry and agriculture, they have also been applied in the biological sciences. Mixture designs can be used for screening complex medium components in the cultivation of bacteria and evaluating the influence of nutrients on bacterial byproducts and growth [26-30]. For instance, Kiviharju et al. [26] apply a mixture design for the screening of suitable complex medium components in the cultivation of *S. peucetius* var. *caesius*, an aerobic bacterium that produces doxorubicin as a secondary metabolite. Rispoli and Shah [27] use mixture experiments to evaluate the influence of six nutrient elements on production of cutinase from the fungus *Colletotrichum lindemuthianum*. For other examples, see [28-30].

We report on an application of the proposed experimental protocol to an analysis of seven vaginally-relevant bacteria and apply the results to clinical samples.

## Results and discussion

### Different DNA extraction kits introduced different bias

We analyzed a single mock community and varied the DNA extraction kit and the number of PCR cycles. The mock community consists of 21 bacterial/archaeal strains from 18 genera [31] that are not necessarily associated with the human vagina. Taxonomic classification was performed using the RDP classifier [32] (see Methods). The choice of DNA extraction kit led to the most striking differences between the protocols tested (Additional file 1). Relative to the Powersoil kit, using the Qiagen kit increased the observed proportion of *Enterococcus* by about 50% while suppressing the observed proportions of *Neisseria*, *Bacillus*, *Pseudomonas*, and *Porphyromonas*. In contrast, changing the number of PCR cycles from 30 to 35 affected the observed proportions only slightly. The small changes due to PCR cycle number agree with previous studies that showed that while chimera formation increases with cycle number, the observed community structure is retained [6,9,10,33,34].

Each combination of extraction kit and cycle number produced results that were dramatically different from the actual mixing proportions. The differences between the observed and actual proportions were different for the different extraction kits. The results for each of the samples produced underestimates of *Lactobacillus* (the only species included in the mock community was *L. gasseri*) and *Streptococcus* (the mock community included *S. pneumoniae*, *S. mutans* and *S. agalactiae*).

### Mixture experiments and mixture effect models for quantifying and characterizing bias in 16S metagenomic studies

A mixture design is an experiment in which a response of interest is assumed to depend only on the relative proportions of the components present in the mixture. If the response changes when the proportions of those components making up the mixture are altered, then the response is said to be a measure of the joint blending property of the components of the mixture [35]. The distinguishing feature of mixture experiments is that the mixture components are subject to a constraint requiring that the proportions sum to one. Due to this constraint on the mixture components, non-standard statistical models are required to model the response. Mixture effect models [36] allow for prediction of the response for given proportions of mixture components as well as evaluation of relationships among the components.

We developed the following protocol for quantifying and characterizing bias in 16S metagenomic studies:

1. Decide upon a small subset of bacteria whose measurement is of interest. We selected seven vaginally-relevant species based on their prevalence in clinical samples, suspected importance in disease mechanisms, and ability to be cultured.
2. Based on the number of bacteria selected and the number of runs available, generate an experimental design. The 80-run mixture experiment for our application was a D-optimal design [37] containing 65 unique treatment combinations and 15 replicate samples. Replicate runs were used to obtain an estimate of the pure error variance.
  The D-optimal design that we used requires at least 63 runs:
  $$\left(7 \atop 3\right) + \left(7 \atop 2\right) + 7 = 63, $$
  in order to fit a special cubic model (see Methods). The 17 additional runs in the design included two other unique treatment combination for testing lack of fit and 15 replicates. With *n* bacteria, the same design would require the number obtained by replacing 7 with *n* in the formula above. For example, an analogous model for 12 bacteria would require a minimum of 298 runs.
3. Randomize the design for three mixture experiments. The treatment combinations and placement on plates were randomized to alleviate effects of bias due to experimental conditions. Each row of the experimental design in Additional file 2 contains a treatment combination that prescribes the proportion of cells, DNA, or PCR product from each strain of bacteria used in the construction of a mock community.
4. Prepare and process mock communities according to the experimental design. Preparing mock communities for each experiment is described below and illustrated in Figure 1.
  Experiment 1. Create mock communities by mixing prescribed quantities of cells from each organism. Grow each isolate to exponential phase and determine cell density through estimates of viable cell counts and optical density; the combined approach improves the accuracy of estimates. Combine bacteria to form mock communities and subject the samples to DNA extraction, PCR amplification, sequencing, and taxonomic classification.

**Figure 1:**
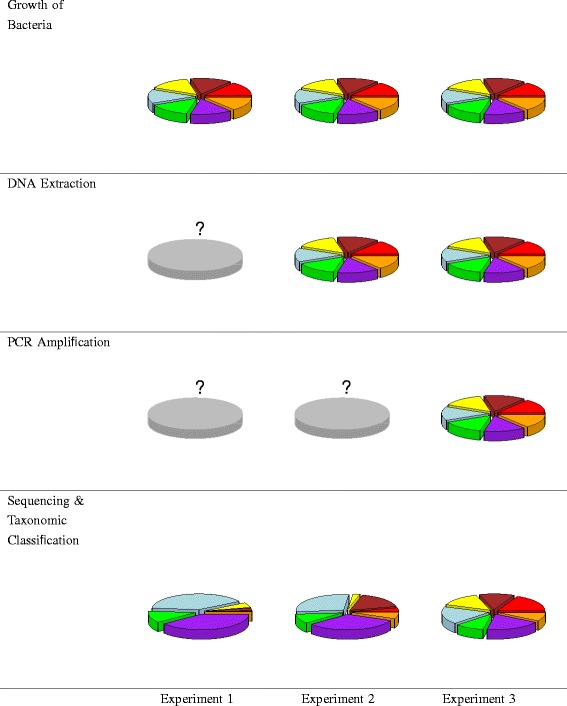
**Schematic of three mixture experiments and observed results.** In Experiment 1, bacterial cultures were mixed so that communities were comprised of equal numbers of cells. In Experiment 2, DNA was extracted from pure bacterial cultures and then mixed so that communities were comprised of equal amounts of DNA. In Experiment 3, DNA was extracted from pure bacterial cultures and subjected to PCR and PCR product was mixed so that communities are comprised of equal amounts of PCR product. The pie charts in the bottom row are the observed results for a sample that consisted of equal proportions of seven bacteria for each experiment. The pie charts in the other rows represent the prescribed mixing ratios (each slice is of equal size). Key: red - *G. vaginalis*, orange - *S. agalactiae*, purple - *S. amnii*,green - *P. bivia*, light blue - *L. iners*, yellow - *L. crispatus*, brown - *A. vaginae*.
  Experiment 2. Create mock communities by mixing proportions of gDNA. Extract gDNA from pure cultures of each bacterial strain. Measure DNA concentration and mix in the proportions described by the experimental design. Then process each sample by PCR amplification, sequencing, and taxonomic classification.
  Experiment 3. Create mock communities by mixing equal proportions of PCR product. Begin by extracting gDNA from the pure cultures of each bacterial species. Subject the pure gDNA to PCR amplification. Mix the PCR products according to the experimental design. Sequence each sample and classify the reads.
5. Compare the differences in the results of each experiment. Comparing the results of Experiment 1 with the prescribed mixing ratios gives a measurement of the total bias. If *x*_*i*_ is the prescribed mixing ratio for bacterium *i* and $\hat {x}_{i}$ is the observed proportion, then the bias is the difference $\hat {x}_{i} - x_{i}$. A negative value indicates that the bacterial signal is suppressed, while a positive value indicates that the signal is amplified.
  Comparing the results of Experiments 1 and 2 isolates the effects of the DNA extraction protocol. Similarly, comparing the results of Experiments 2 and 3 isolates the effects of bias due to the PCR amplification protocol. Comparing the results of Experiment 3 with the prescribed mixing ratios isolates the effects of sequencing and taxonomic classification. The pie charts at the bottom of Figure 1 show the results for a sample that contained equal proportions of the seven bacteria for each of our experiments. Similar information would be generated for each mock community in each experiment, facilitating analysis via modeling.
6. Fit mixture effect models to regress the observed proportions of reads against functions of the prescribed mixing ratios. Significant terms in the models can reveal pairs or groups of bacteria that, when present together in a sample, amplify or suppress the observed proportions of other bacteria.
7. Fit models to regress the prescribed mixing ratios against the observed proportions of reads for prediction of actual community composition based on the observed community composition. Use cross-validation to estimate accuracy for clinical/environmental samples.

In the sections that follow, we report on the results of our application of this protocol for seven species: *Atopobium vaginae*, *Gardnerella vaginalis*, *Lactobacillus crispatus*, *Lactobacillus iners*, *Prevotella bivia*, *Sneathia amnii*, and *Streptococcus agalactiae*.

### Contaminating bacteria did not significantly contribute to bias in small mock communities

A total of 3.9 million reads were generated for 240 samples across three experiments involving seven vaginally-relevant bacteria. Taxonomic classification was performed using the STIRRUPS method and reference database [38]. Only 2,279 (<0.06%) below-threshold reads and 733 (<0.02%) above-threshold reads were assigned to taxa not in the study. None of the samples had large proportions of reads assigned to taxa not in the study (third quartile 0.02%, max 2.8%).

The three experiments consisted of mixing live bacteria, extracted DNA, and PCR product according to prescribed proportions (Additional file 2). Of the 80 samples in each experiment, 15 were technical replicates; i.e., there were 65 unique mixtures, 15 of which were repeated. The median absolute error, a measure of the technical variation among replicates, of the observed proportion of each organism was largest for the experiment mixing prescribed quantities of PCR product; the median absolute error was smallest for mixing live bacteria (Additional file 3). For all bacteria and experiments, the median absolute error was less than 5%. Therefore, technical variation was not a confounding factor in our results.

### Our DNA extraction and PCR amplification protocols contributed more to bias than sequencing error and taxonomic misclassification

The results when mixing prescribed proportions of live bacteria, extracted DNA, and PCR product can be used to isolate the effects of the DNA extraction protocol, the PCR amplification protocol, and sequencing error and taxonomic classification.

Bacteria were collected from late log cultures and correlated with OD _600 nm_ to minimize the number of non-viable bacteria within the cultures. However, there was likely some contribution of DNA from non-viable bacteria and this would be expected to contribute to the bias. In these experiments, bias due to differing numbers of non-viable bacteria would not be distinguishable from bias due to our DNA extraction protocol. Throughout the remainder of the paper, it is implied that the bias due to DNA extraction includes bias due to non-viable bacteria.

**Figure 2:**
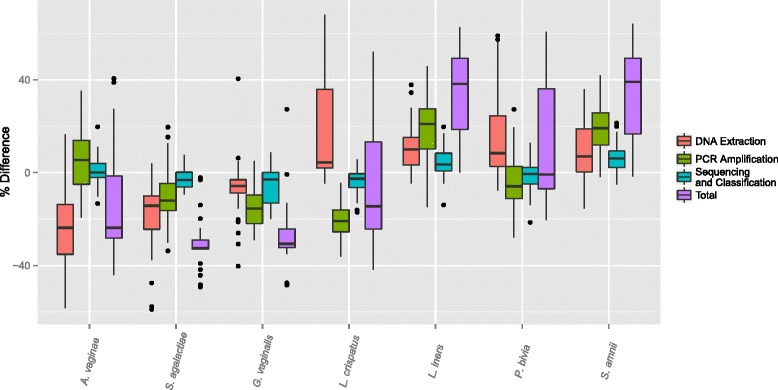
**Observed bias by bacterium.** The observed bias (the observed minus the actual proportions) for each bacterium in the experimental design due to the different effects of our DNA Extraction, PCR amplification, and sequencing and taxonomic classification protocols. The total bias is also plotted for each bacterium. For each box and whisker plot, only the samples including the bacterium were included.

A box plot of the bias for all mixtures for each of the three experiments and for each of the seven bacteria is plotted in Figure 2. The bias due to sequencing and classification was smallest, as indicated by the fact that the median bias was between -5% and 5% for each bacterium, and by the small inter-quartile ranges. The bias due to our DNA extraction protocol and our PCR amplification protocol contributed the most to total bias. Our observation that our PCR amplification protocol contributed more to bias than sequencing-specific causes confirms the results of a previous study [8]. Sequencing and classification error effects were likely reduced in our experiment because of the small numbers of bacteria in samples. Also, the use of a carefully-curated database and species-level classification method [38] likely helped to filter chimeric sequences.

### The effects of our DNA extraction and PCR amplification protocols were dependent on bacteria

The inter-quartile ranges for bias from our DNA extraction protocol indicated that our protocol amplified the observed proportions of *L. crispatus*, *L. iners*, *P. bivia*, and *S. amnii* while suppressing those of *A. vaginae*, *S. agalactiae*, and *G. vaginalis*. The same analysis for our PCR amplification protocol shows that the observed proportions of *A. vaginae*, *L. iners*, and *S. amnii* were increased while those of *S. agalactiae*, *G. vaginalis*, *L. crispatus*, and *P. bivia* were decreased. With the exception of bias due to our PCR amplification protocol and sequencing and classification for *A. vaginae* and *P. bivia*, the bias was significantly different from zero (*p*<0.05).

The effect of each processing step (DNA extraction, PCR amplification, sequencing and taxonomic classification) was dependent on the bacterium. The bias due to the different processing steps appeared to be independent because the bias from each step was cumulative and was reflected in the results for the total bias for each bacterium. The total bias is the observed proportion minus the proportion of cells included in the mixture in the first experiment. Overall, the observed proportions of *A. vaginae*, *S. agalactiae*, *G. vaginalis*, and *L. crispatus* was less than the proportions of bacteria in the mixtures, and the observed proportions of *L. iners* and *S. amnii* was more than the proportions in the mixtures. The inter-quartile range for total bias for mixtures containing *P. bivia* was large and contains zero, indicating that the bias was affected by which particular bacteria were also included in a mixture. The median total bias for *S. agalactiae* was -32.6%, indicating that in a mixture containing *S. agalactiae*, we would expect to observe that its proportion would be 32.6% less than its true representation in the community. The largest positive median bias among the bacteria was 39.1% for *S. amnii*.

Previous studies observed that the copy number and genome size may not be consequential sources of bias [19,33]. The analysis here appears to agree that these factors were not sufficient to describe bias in the PCR step. For example, *S. agalactiae* had the largest copy number among the organisms in this experiment, but the observed proportions were consistently less than the actual proportions in the mock communities.

### Observed proportions of bacteria were amplified or suppressed by the presence of other bacteria

The interquartile range for bias due to our DNA extraction protocol was larger than that for bias due to our PCR amplification protocol or sequencing and classification for all but one bacterium (Figure 2), but the technical variation was largest when mixing pure PCR product (Additional file 3). Therefore, the wider ranges of bias due to our DNA extraction protocol was likely due to some relationship between bacterial signals. Here we present a deeper analysis of the effects observed at each step.

In traditional mixture experiment terminology, an *interaction* is a causative effect in the observed proportions of bacteria that may or may not reflect a physical relationship between the bacteria. The effect could be a result of a difference in proclivity to use resources. For example, one bacterium could yield more sequences in PCR product than another because of template re-annealing or primer design. For a bacterium A, there is a *synergistic interaction* with bacterium B if the presence of bacterium B increases the observed proportion of bacterium A. Likewise, there is an *antagonistic interaction* with bacterium B if the presence of bacterium B decreases the observed proportion of bacterium A. Because the term “interaction” is often interpreted in common use to connote a physical effect, we attempt to avoid the confusion and use the terms “relationship”, “synergistic relationship”, and “antagonistic relationship”.

Comparison of the prescribed proportions of bacteria with the results of the experiment mixing live bacteria can be used to evaluate whether the observed proportions of bacteria are promoted or suppressed by the presence of other bacteria. Special cubic mixture effect models, where the dependent variable is the observed proportion of a bacterium, reveal statistically significant blends of bacteria (Additional file 4). Each model has linear terms that capture the main effects of having bacteria present in the sample along with quadratic and cubic blending terms that capture higher-order relationships.

The model fits were clearly strong as indicated by *R*^2^ values above 0.99 for each model. As expected from the results in Figure 2, there were far fewer statistically significant blending terms for the models based on mixing equal amounts of PCR product than for those based on mixing equal numbers of cells or those based on mixing equal quantities of DNA (Table 1, Additional file 4). The binary blends tended to be more statistically significant than ternary blends. The number of significant blending terms decreased for each subsequent experiment, as would be expected because the sources of bias due to our DNA extraction and PCR amplification protocols were removed. The interaction terms that were significant for the models for mixing cells (Experiment 1) but not significant for the models for mixing DNA (Experiment 2) highlight relationships for our DNA extraction protocol. Similarly, those terms that were significant for the models for mixing DNA (Experiment 2) but not significant for the models mixing PCR product (Experiment 3) highlight relationships for our PCR amplification protocol.

**Table 1:** **The number of significant blending terms (** ***p*** **< 0.05) for each mixture effect model**

**Bacterium**	**Experiment 1**	**Experiment 2**	**Experiment 3**
	**Mixing cells**	**Mixing DNA**	**Mixing PCR product**
*A. vaginae*	14	10	2
*G. vaginalis*	16	8	3
*L. crispatus*	18	6	4
*L. iners*	15	10	3
*P. bivia*	14	7	3
*S. agalactiae*	12	14	3
*S. amnii*	18	12	1

The *p*-values were adjusted using the Bonferroni correction.

**Figure 3:**
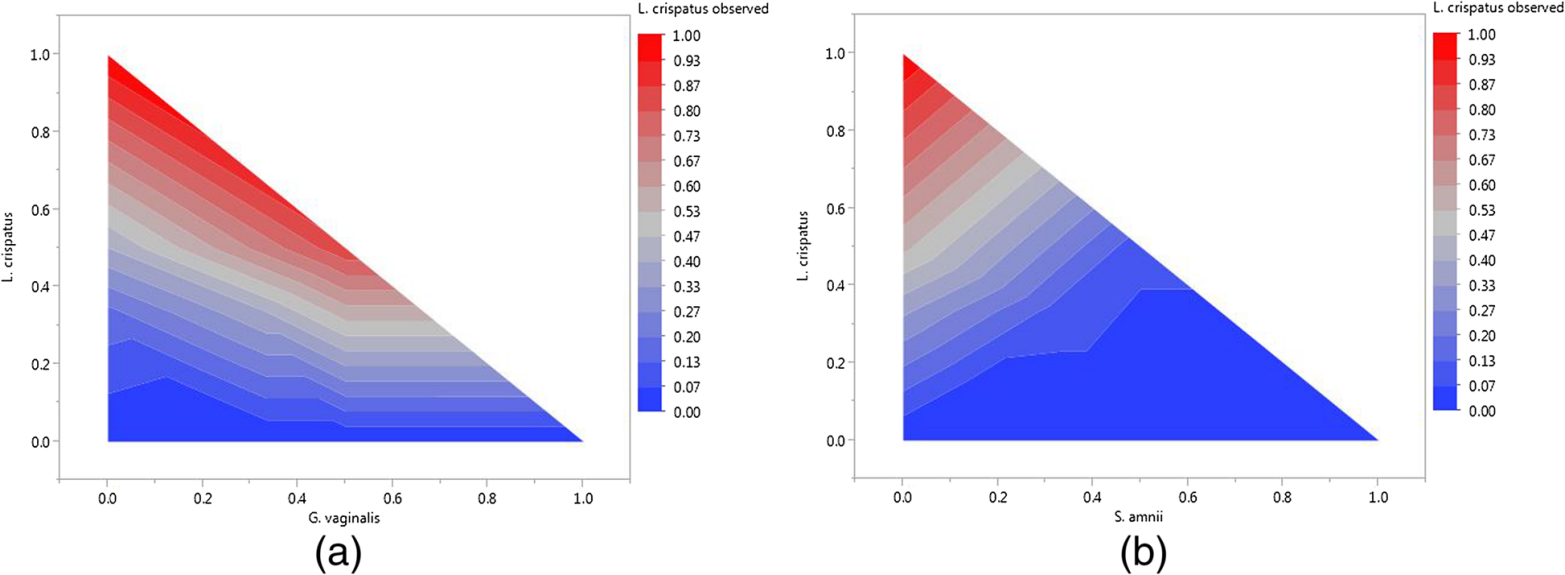
**Interaction/blending plots for** ***L. crispatus*** and (a) ***G. vaginalis*** **and (b)** ***S. amnii*** **.** The contours indicate the expected observed amount of *L. crispatus* for a given actual percentage of a sample for a pair of bacteria.

The models for the experiment mixing cells generated interaction/blending plots as shown in Figure 3. As an example, Figure 3(a) depicts the blending of *L. crispatus* and *G. vaginalis*. For a given concentration of *L. crispatus*, as more *G. vaginalis* is added to a sample (keeping absolute input quantities from other bacteria constant), the expected proportion of observed *L. crispatus* will increase. Therefore, *G. vaginalis* interacts synergistically with *L. crispatus*. The hypoteneuse of the triangle reflects the expected results when *L. crispatus* and *G. vaginalis* are the only bacteria in a mixture. The shaded contours if the remainder of the triangle indicate the expected observed *L. crispatus* proportion, averaging over the effects of including the other bacteria in the experiment at proportions resulting in a mixture that sums to 100%. The figure, therefore, depicts the expected relationship between two bacteria when included in blends at different levels.

Figure 3(b) depicts the antagonistic blending of *S. amnii* with *L. crispatus*: as more *S. amnii* are added to a sample with *L. crispatus*, the expected proportion of observed *L. crispatus* will decrease. These plots reflect properties that were quantified for statistical significance in the models (Additional file 3). For the model based on mixing equal numbers of cells, for which the observed proportion of *L. crispatus* is the dependent variable, the binary blend with *G. vaginalis* was significant with a coefficient estimate of 1.41, indicating that if *L. crispatus* and *G. vaginalis* were present in a sample together, then the proportion of observed *L. crispatus* would larger than what is truly present. This term in the model contributes 1.41 times the product of the actual proportions of *L. crispatus* and *G. vaginalis* to the expected amount of *L. crispatus* observed. For the same model, the binary blend for *L. crispatus* and *S. amnii* was significant with a negative coefficient estimate (-1.66), indicating that the observed proportion of *L. crispatus* decreases with increases in the proportion of *S. amnii*. If we compare this model with the model for mixing DNA, we see that the binary blend with *G. vaginalis* was not significant, but the binary blend with *S. amnii* was significant. These results indicated that during our DNA extraction process, the observed amount of *L. crispatus* will be increased relative to *G. vaginalis* and decreased relative to *S. amnii*. During our PCR amplification process, there was additional bias decreasing the observed amount of *L. crispatus* relative to *S. amnii*, but no significant relationship with *G. vaginalis* existed at this step.

Additional file 5 depicts the ratio of the observed *L. crispatus* to actual *L. crispatus* versus the proportion of *G. vaginalis* and *S. amnii*. The trend lines and the departures from *y*=1.0 help to indicate the blending effect. The wide variation in the data around the model indicated that the identities of the additional bacteria in a mixture were important for predicting the observed proportions of *L. crispatus*. Tables 2 and 3 contain the statistically significant synergistic and antagonistic binary blends for the experiment based on mixing equal numbers of cells. The significant relationships are ordered by decreasing significance. Nearly all of the bacteria were synergistic with *L. iners* and *S. amnii*, and nearly all are antagonistic with *G. vaginalis*. *G. vaginalis* was synergistic with all bacteria except *S. agalactiae*. All bacteria were antagonistic with *S. agalactiae*. These results indicate that the observed proportions of *L. iners* and *S. amnii* in samples were overestimates while those for *G. vaginalis* and *S. agalactiae* were underestimates.

**Table 2:** **Significant synergistic binary blends ordered by effect size**

**Bacterium**	**Synergistic relationships**
*L. crispatus*	*S. agalactiae, G. vaginalis, A. vaginae*
*G. vaginalis*	*S. agalactiae*
*A. vaginae*	*S. agalactiae, G. vaginalis*
*L. iners*	*S. agalactiae, L. crispatus, G. vaginalis, A. vaginae, P. bivia*
*P. bivia*	*A. vaginae, S. agalactiae, G. vaginalis, L. crispatus*
*S. amnii*	*S. agalactiae, L. crispatus, G. vaginalis, A. vaginae, P. bivia*
*S. agalactiae*	None

**Table 3:** **Significant antagonistic binary blends ordered by effect size**

**Bacterium**	**Antagonistic relationships**
*L. crispatus*	*S. amnii, L. iners, P. bivia*
*G. vaginalis*	*L. iners, S. amnii, P. bivia, L. crispatus, A. vaginae*
*A. vaginae*	*P. bivia, L. iners, S. amnii, L. crispatus*
*L. iners*	None
*P. bivia*	*L. iners, S. amnii*
*S. amnii*	None
*S. agalactiae*	*L. crispatus, S. amnii, L. iners, P. bivia, A. vaginae, G. vaginalis*

**Figure 4:**
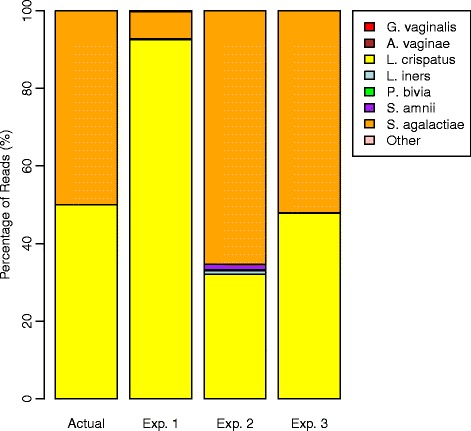
**Results for mixture of** ***L. crispatus*** **and** *S. agalactiae* **.** Actual and observed proportions of bacteria when mixing equal proportions of cells (Exp. 1), DNA (Exp. 2), and PCR product (Exp. 3) for *L. crispatus* and *S. agalactiae*.

Figure 4 presents the results of binary blends of equal amounts of cells, DNA, and PCR product for *L. crispatus* and *S. agalactiae*. When equal numbers of cells were mixed, over 92.5% of reads were assigned to *L. crispatus*, which is an error of 85%. When equal amounts of DNA were mixed, only 32% were assigned to *L. crispatus*. The discrepancy indicates that our DNA extraction protocol tends to increase the proportion of *L. crispatus* reads observed over that of *S. agalactiae*. The opposite trend was observed when comparing the results of mixing equal amounts of DNA and equal amounts of PCR product: the signal for *S. agalactiae* was increased and the signal for *L. crispatus* decreased during PCR. When equal amounts of PCR product were mixed, the observed proportions of the two bacteria were nearly equal, indicating that sequencing and taxonomic misclassification error did not contribute to observed bias.

Among most samples with equal amounts of cells, rank abundance in the biased results was preserved. In other words, if for a pair of bacteria present in a sample the first is observed to comprise a larger proportion than the second, then the observed proportion for the first bacterium is larger than the second in most of the other samples containing those bacteria. A notable exception is depicted in Table 4. Sample 1 contained equal proportions of *S. amnii*, *L. iners*, and *S. agalactiae*. Because of bias, more *S. amnii* than *L. iners* was observed in Sample 1 in Table 4, and very little *S. agalactiae* was detected. Sample 2 also contained equal proportions of *S. amnii* and *L. iners* along with *G. vaginalis* and *P. bivia*. For this sample, more *L. iners* was observed than *S. amnii*. The differences were larger than the technical variation depicted in Additional file 2. Therefore, rank abundance was not preserved for *L. iners* and *S. amnii*. It is not clear whether the result was due to the larger number of bacteria in the samples than other samples, which is more representative of clinical and environmental samples, or was a reflection of tertiary and higher-order effects between combinations of bacteria.

**Table 4:** **Actual and observed proportions of bacteria for two mock community samples containing** ***L. iners*** **and** ***S. amnii***

	**Sample 1**		**Sample 2**	
**Bacteria**	**Observed**	**Actual**	**Observed**	**Actual**
*A. vaginae*	0.0%	0.0%	0.0%	0.0%
*G. vaginalis*	0.0	0.0	0.8	25.0
*L. crispatus*	0.0	0.0	0.0	0.0
*L. iners*	47.2	33.3	48.7	25.0
*P. bivia*	0.0	0.0	18.7	25.0
*S. amnii*	52.3	33.3	31.8	25.0
*S. agalactiae*	0.4	33.3	0.0	0.0

### Mixture effect models predicted community composition in clinical samples

**Figure 5:**
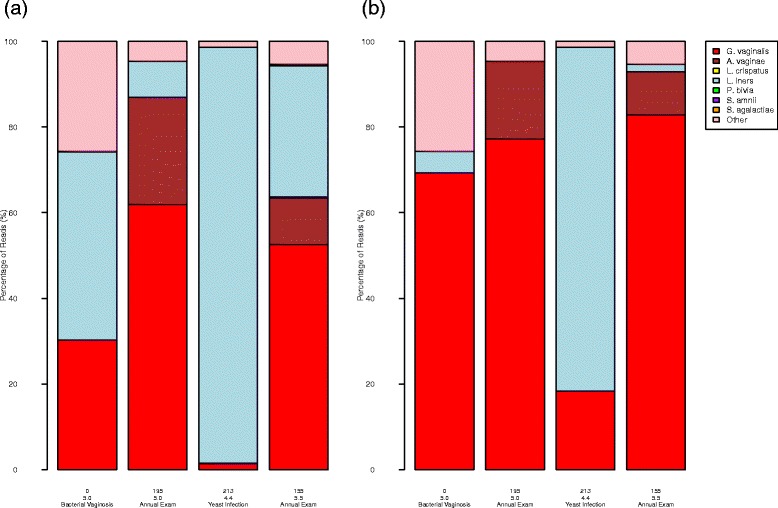
**(a) Observed and (b) predicted proportions of bacteria of four clinical samples.** The samples are from the same subject in different visits.

Models constructed via an inverse fit (i.e., treating the mixing proportions of bacteria in a sample as the dependent variable) can be applied to the observed proportions of bacteria in clinical samples to estimate the true community composition. Figure 5 depicts the observed and predicted proportions of bacteria for samples from the mid-vaginal wall of a subject over the course of four visits to a clinic. Low diversity and richness in vaginal samples, such as that observed for this subject, is not unusual [39].

Because of the low diversity, vaginal samples are often classified by the predominant bacterium into community states or types [40]. The first sample, unadjusted, would be categorized as being of the *L. iners* type. However, the inverse models predicted that the true composition of the bacterial community was dominated by *G. vaginalis*. In our analysis of samples of the *L. iners* type from other subjects, we often observed subgroups consisting of those with nontrivial levels of *G. vaginalis* as in sample 1 and those with little as in sample 3 (unpublished results).

The bacteria common to the clinical samples and the mixture effect experiments were *G. vaginalis*, *L. iners*, and *A. vaginae*. *G. vaginalis* is often associated with a diagnosis of bacterial vaginosis (BV) and an elevated pH. *L. iners* and *A. vaginae* are lactic acid-producing bacteria known to lower pH. The observed proportion of *G. vaginalis* was larger for sample 2 than sample 4 and the proportion of lactic acid-producing bacteria was smaller, yet the pH was higher for sample 4. The predicted proportions aligned better with the pH measurements. Sample 3 was predicted to have the highest proportion of lactic acid-producing bacteria and was associated with the lowest pH, samples 1 and 2 had similar proportions of *G. vaginalis* and were associated with the same pH, and sample 4 had the largest proportion of *G. vaginalis* and was associated with the highest pH. Though samples 2 and 4 had the highest proportions of *G. vaginalis* and the highest measured pHs, there was no diagnosis. The lack of symptoms and/or disease may have been due to the presence of *A. vaginae*.

## Conclusions

We have demonstrated that models based on analysis of small mock communities can enhance our understanding of bias and the analysis of low-diversity environments such as the human vagina. The same protocol could be applied to bacteria from more diverse environments such as the human gut or soil samples to understand how the most dominant species are affected by bias. As demonstrated here, a good understanding of bias can change and improve the conclusions based on the analysis of clinical or environmental samples.

We recommend that labs use small mock communities for understanding the effects of bias for their particular choices of protocols. Fitting mixture effect models is useful for establishing statistical significance concerning the relationship between observed proportions for pairs of bacteria. Though our results from a full mixture experiment and mixture effect models were informative, it is perhaps excessive for validating a pipeline. Smaller sets of mock communities, with around 10-20 blends of bacteria, are likely sufficient for labs to assess the effects of bias for certain taxa so that analysis may be qualitatively hedged. For example, if our lab observes clinical samples with both *L. iners* and *G. vaginalis*, we may presume that *L. iners* is likely over-estimated and *G. vaginalis* is under-estimated.

Additional experiments with axial blends and blends of larger numbers of bacteria are needed to understand if rank abundance is preserved. Using the protocol presented here to model bias for more diverse environments would require the creation and analysis of an impracticable number of mock communities because of the number of bacteria that need to be modeled. Therefore, it is necessary to further understand the factors contributing to bias from DNA extraction and PCR amplification protocols such as cell lysability, primer efficiency, Gram negativity, and GC content. Models based on these factors, along with careful curation of reference databases containing such information, could enhance the interpretation of results from studies of diverse communities.

The results presented here indicate that bias due to our DNA extraction and PCR amplification protocols are much greater than the effects of sequencing and taxonomic classification. Therefore, we can expect that bias will remain a challenge even as sequencing technology advances. The effects of bias can lead to the discovery of spurious correlations (linear relationships) and to missed true correlations. The results of ground-truthing with small mock communities can help to hedge conclusions obtained by analyzing observed relative quantities. Efforts to assess bias within labs as proposed here and guidelines for best practices across labs, a goal of The Microbiome Quality Control project [3], will facilitate the extraction of more useful information from experiments in various domains.

## Methods

### Mock community preparation and processing

The seven strains used, 16S rRNA gene copy numbers, and genome sizes are in Table 5. Copy numbers and genome sizes were estimated from NCBI [41]. *Atopobium vaginae*, *Gardnerella vaginalis*, *Lactobacillus iners*, *Prevotella bivia*, *Streptococcus agalactiae*, and *Sneathia amnii* were cultivated on Brain Heart Infusion (BHI) agar plates (EMD, Gibbstown, NJ) supplemented with 1% yeast extract, 2% gelatin, 0.1% starch, 0.1% glucose, and 10% human blood (sBHI) or in sBHI broth containing 10% human blood instead of serum [42]. *Lactobacillus crispatus* was grown on De Man Rogosa Sharpe (MRS) agar plates or in MRS broth. All bacteria were cultured at 37°C under anaerobic conditions (AnaeroPack, Mitsubishi Gas Chemical Co, Tokyo, Japan) until they reached late log phase. The optical density at 600 nm was determined and the bacteria were enumerated by counting colonies on solid medium. OD _600 nm_ values and colony forming units (CFUs) were determined in three separate experiments to ensure that the correlation between OD _600 nm_ values and CFU was precise for each species. Bacteria were aliquoted and kept frozen at -80°C until use. Bacteria were combined at the prescribed proportions based on CFU. DNA was extracted from the combinations using the PowerSoil DNA Isolation Kit from MO BIO Laboratories, Inc. (Carlsbad, California) and 2 *μ*L of DNA was used in each PCR reaction.

**Table 5:** **Strains used in experimental design and modeling**

**Species**	**Genome size**	**Copy number**	**Gram +/-**
*A. vaginae*	1.43	1	+
*G. vaginalis*	1.65	2	+
*L. crispatus*	2.04	4	+
*L. iners*	1.30	1	+
*P. bivia*	2.47	1	-
*S. agalactiae*	2.20	7	+
*S. amnii*	1.34	1	-

For the DNA combinations, DNA was isolated from approximately 1×10^10^ bacteria using the Qiagen Genomic Tip 100/G kit (Valencia, CA) and DNA concentration and purity was measured using a Nanodrop (Thermo Fisher). The Qiagen kit was used only for extractions from pure cultures; using a different kit for these extractions would not create bias because the DNA are mixed at prescribed proportions after the extractions. The DNA from different species was combined to produce a final DNA concentration of 2 ng/ *μ*L, and 2 *μ*L of these dilutions were used in each PCR reaction.

For the PCR combinations, 4 ng of DNA from each organism was amplified by PCR and the PCR reactions were combined based on volume, according to the prescribed combinations.

The protocols of the Vaginal Human Microbiome Project for 16S rRNA gene sequencing have been previously described [38,43], and were followed for this study. The V1-V3 hypervariable regions of the bacterial 16S rRNA gene were amplified by PCR using barcoded primers. For each reaction, 4 ng of DNA was combined with 33 *μ*L PCR Supermix High Fidelity™, 11 *μ*L Platinum PCR Supermix™ (Life Technologies), and 100 nM each of forward and reverse primers. The 16S primers contain the A or B Titanium sequencing adapter (shown in italics), followed immediately by a unique variable (6-9 base) barcode sequence and finally the 20 nucleotide sequence complementary to the targeted region of the 16S rRNA gene. The forward primer was a mixture (4:1) of the primers Fwd-P1 (5’ - *CCATCTCATCCCTGCGTGTCTCCGACTCAG* BBBBBB AGAGTTYGATYMTGGCTYAG) and Fwd-P2 (5’ - *CCATCTCATCCCTGCGTGTCTCCGACTCAG* BBBBBB AGARTTTGATCYTGGTTCAG). The reverse primer was Rev1B (5’ – *CCTATCCCCTGTGTGCCTTGGCAGTCTCAG* ATTACCGCGGCTGCTGG). PCR products were sequenced using the Roche 454 GS FLX Titanium platform. The forward PCR primer is a mix of 20 different primers corresponding to positions 8 to 27 of the *E. coli* 16S rRNA genes. This primer mix contains primers that perfectly match the taxa used in this study with the exception of *G. vaginalis* with a single mismatch at position 19 (A/G) from the 3’-end of the forward primer.

Observed counts for the experiments mixing equal proportions of live bacteria were adjusted by dividing by 16S rRNA gene copy number. Observed counts for the experiments mixing equal proportions of DNA were adjusted by multiplying by genome size and dividing by copy number. Counts in each experiment were normalized to proportions.

The raw sequence data are available at http://www.ncbi.nlm.nih.gov/bioproject/267701 under BioProject ID PRJNA267701.

### Species-level taxonomic classification

For both mock community samples and clinical samples, we processed reads for which valid primer and multiplex identifier sequences were observed, less than 10% of base calls had a quality score less than 10, the average quality score was greater than Q20, and the read length was between 200 and 540 bases. The reads were not trimmed. The STIRRUPS method for species-level taxonomic classification was used as previously described [38]. Sequences that aligned with at least 97% global sequence identity to a sequence in the reference database were classified. Across the three experiments, 253,078 reads (6.5%) were below threshold and were not included in subsequent analysis. Of the below-threshold reads, 252,796 were assigned to taxa in the study.

### Experimental design and mixture effect models

With a mixture experiment, the levels of the individual bacteria strains cannot be set independently because the community proportions must sum to one. Suppose there are *p* bacteria and *x*_*i*_ denotes the proportion of the *i*^*th*^ bacterium in a mock community. Then, the *x*_*i*_s are constrained such that *x*_*i*_≥0 for *i*=1,2,…,*p* and that

$$\sum_{i=1}^{p} x_{i} = 1. $$

Due to the constraint that each treatment combination must sum to one, the form of mixture effect polynomials is somewhat different from the standard polynomials used in response surface modeling. In particular, we made use of Scheffé mixture effect models [36]. It is often the case in mixture experiments that a higher-order response surface model is needed to adequately model the response. We used a special cubic model of the form

$${} E(y) = \sum_{i=1}^{p} \beta_{i} x_{i} + \sum_{i=1}^{p-1} \sum_{j=2}^{p} \beta_{ij}x_{i}x_{j} + \sum_{i=1}^{p-2}\sum_{j=2}^{p-1}\sum_{k=3}^{p} \beta_{ijk}x_{i}x_{j}x_{k}. $$

Each coefficient *β*_*i*_ represents the expected response when *x*_*i*_=1 and all other components are zero (i.e., pure blends). The *β*_*ij*_ and *β*_*ijk*_ are binary and ternary blending coefficients. For instance, each *β*_*ij*_ is a measure of the departure from linearity when bacteria *i* and *j* are blended together. The sign of the nonlinear blending coefficients indicate whether the relationship is synergistic (positive coefficient) or antagonistic (negative coefficient). Models were fit using JMP [46]. Additional file 6 contains a JMP scripting language (JSL) script for fitting mixture effect models.

### Quantifying contribution to bias

The differences in Figure 2 between observed and actual proportions were included only for mixtures in which the bacteria were present.

To test for statistical significance of bias, samples were matched between pairs of experiments (Experiments 1 and 2, 2 and 3, and 3 and the prescribed mixing ratios). Bootstrap confidence intervals for the Mahalanobis distance were calculated for each pair, and significance was established if zero was in the confidence interval. The Mahalanobis distance is a measure that accounts for correlations in the data so that distances along directions of low variation are larger and vice versa. Figures were generated and tests conducted using the R Environment for Statistical Computing and the packages *bootstrap* [44] and *ggplot2* [45]. R code and a guide to data and scripts is contained in Additional file 7. Additional data and scripts are contained in Additional files 8, 9, 10, 11, 12, 13, 14 and 15.

### Clinical samples and inverse models

Clinical samples were collected as part of the Vaginal Human Microbiome Project (VaHMP). Protocols for subject enrollment and sample collection have been previously described [38,43].

Inverse models were constructed to predict actual proportions based on observed proportions of bacteria in samples where equal quantities of cells were mixed. The dependent variable in these models is a scaling factor: the observed proportion divided by the actual proportion (observations are created for nonzero actual proportions only). The predictors were the actual proportions in each sample. One model was constructed for each bacterium. The models were used to make predictions of scaling factors for bacteria in clinical samples with non-zero counts. The observed values were adjusted for copy number and then multiplied by the predicted scaling factors to yield predictions for the actual proportions. These predictions were re-normalized to sum to 100%. Each model was a random forest model built with 500 trees and two splitting variables in each tree using the R package randomForest [47]. R code is contained in Additional file 7.

### Processing of jumpstart consortium mock community

The composition and analysis of the Human Microbiome Project Jumpstart Consortium Mock Community have been previously described [18,31]. DNA Extraction, PCR amplification, and taxonomic sequencing were conducted as described for the other experiments. Taxonomic classification was performed using the Ribosomal Data Project Classifier [32] with a confidence threshold of 80%.

## Additional files

Additional file 1
**Comparison of DNA extraction kits and number of PCR cycles.** Stacked bar plot of observed proportions of bacteria for a mock community consisting of 21 strains of bacteria when using PowerSoil and Qiagen DNA extraction kits and when allowing 30 and 35 PCR cycles.

Additional file 2
**Experimental design.** Table of the prescribed mixing proportions, plate, and barcode for the experiments mixing equal proportions of cells, DNA, and PCR product.

Additional file 3
**Plot of technical variation in observed proportions of bacteria.** Boxplot of the technical variation, measured as the absolute difference between observed proportions in replicate samples, for each bacterium and for experiments mixing equal proportions of cells, DNA, and PCR product.

Additional file 4
**Tables of significant blending terms for mixture effect models.** Coefficient estimates, *t* ratios, and *p* values for significant cofficients of mixture effect models for each bacterium and for experiments mixing equal proportions of cells, DNA, and PCR product.

Additional file 5
**Alternate visualization of Figure** 3 **with data.** (left) The ratio of observed to actual *L. crispatus* versus the actual *G. vaginalis* for the samples, and the expected values based on mixture effect models. (right) The ratio of observed to actual *L. crispatus* versus the actual *S. amnii* for the samples, and the expected values based on mixture effect models.

Additional file 6
**JMP Scripting Language file.** Script for fitting mixture effect models for the experiments mixing equal amounts of cells, DNA, and PCR product.

Additional file 7
**Code, comments, and output of analysis in R.** R code, comments, figures, and results for analyzing the data from the experiments mixing equal amounts of cells, DNA, and PCR product. Created using R Markdown and knitr. The code demonstrates the use of Additional file 8 through Additional file 14.

Additional file 8
**Taxonomic classification of reads.** Taxonomic classification of reads from experiments mixing equal amounts of cells, DNA, and PCR product. This file is the output of the STIRRUPS [38] pipeline.

Additional file 9
**Python script for creating counts tables.** Python script for creating tables of above-threshold and below-threshold counts for the reads from the experiments mixing equal amounts of cells, DNA, and PCR product.

Additional file 10
**Table of above-threshold counts.** Above-threshold counts for each sample in the experiments mixing equal amounts of cells, DNA, and PCR product.

Additional file 11
**Table of below-threshold counts.** Below-threshold counts for each sample in the experiments mixing equal amounts of cells, DNA, and PCR product.

Additional file 12
**Design and results for experiment mixing cells.** Prescribed mixing proportions and observed proportions for the experiment mixing equal numbers of cells.

Additional file 13
**Design and results for experiment mixing DNA.** Prescribed mixing proportions and observed proportions for the experiment mixing equal amounts of DNA.

Additional file 14
**Design and results for experiment mixing PCR product.** Prescribed mixing proportions and observed proportions for the experiment mixing equal amounts of PCR product.

Additional file 15
**File containing information about the classification of each read for the experiments mixing equal amounts of cells, DNA, and PCR product.** The columns are sample name, read name, RDP classification, RDP score, RDP classification level, STIRRUPS species classification, above threshold (AT) or below threshold (BT) STIRRUPS classification, STIRRUPS score.
