# Supplementary figures and images for: The truth about metagenomics: quantifying and counteracting bias in 16S rRNA studies
Source: BMC Microbiol. 2015 Mar 21;15:66. doi: 10.1186/s12866-015-0351-6 (PMC4433096; doi:10.1186/s12866-015-0351-6)

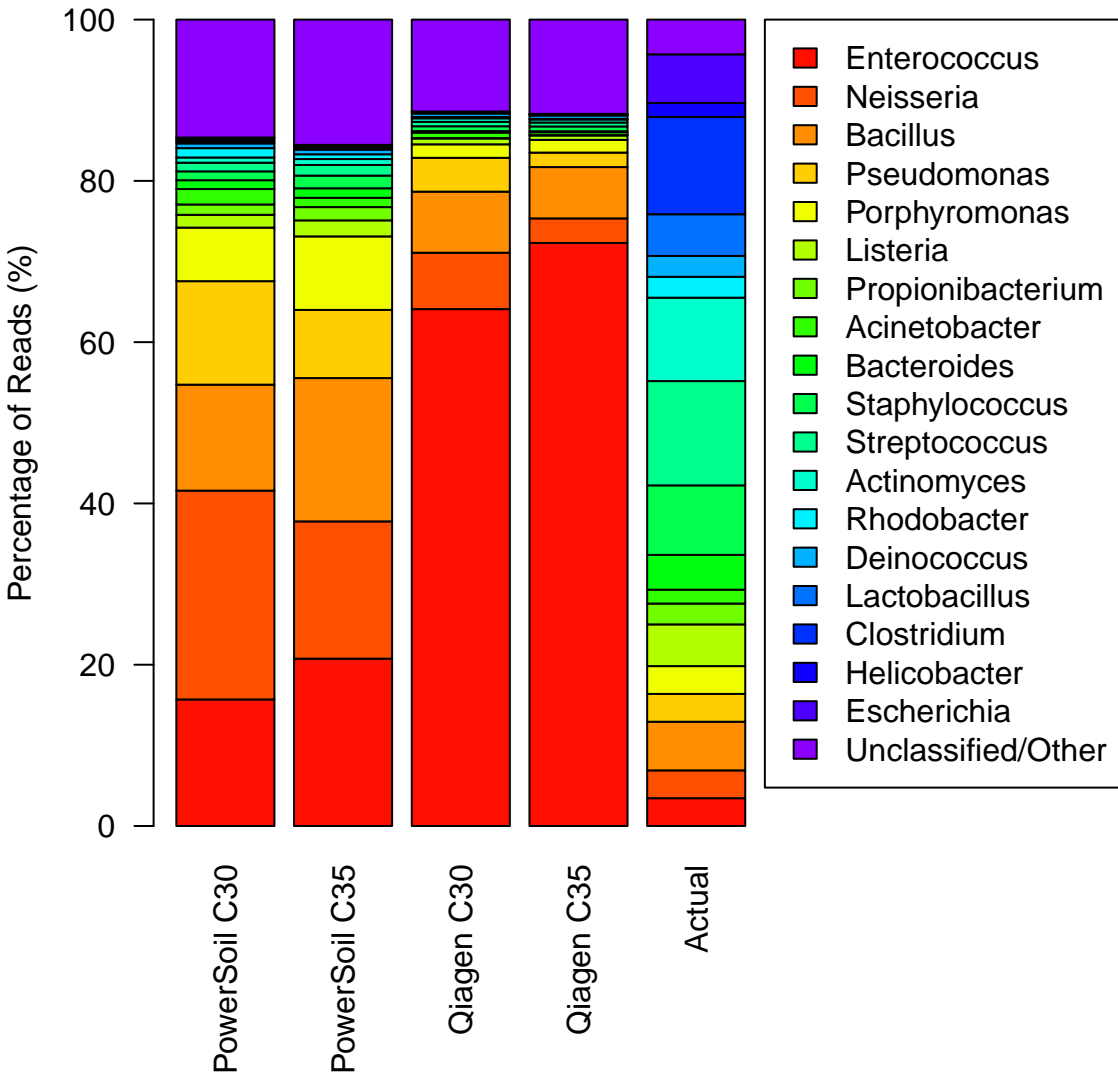

Supplement: Additional file 1 — Comparison of DNA extraction kits and number of PCR cycles. Stacked bar plot of observed proportions of bacteria for a mock community consisting of 21 strains of bacteria when using PowerSoil and Qiagen DNA extraction kits and when allowing 30 and 35 PCR cycles. [file 12866_2015_351_MOESM1_ESM.pdf]

Absolute Error

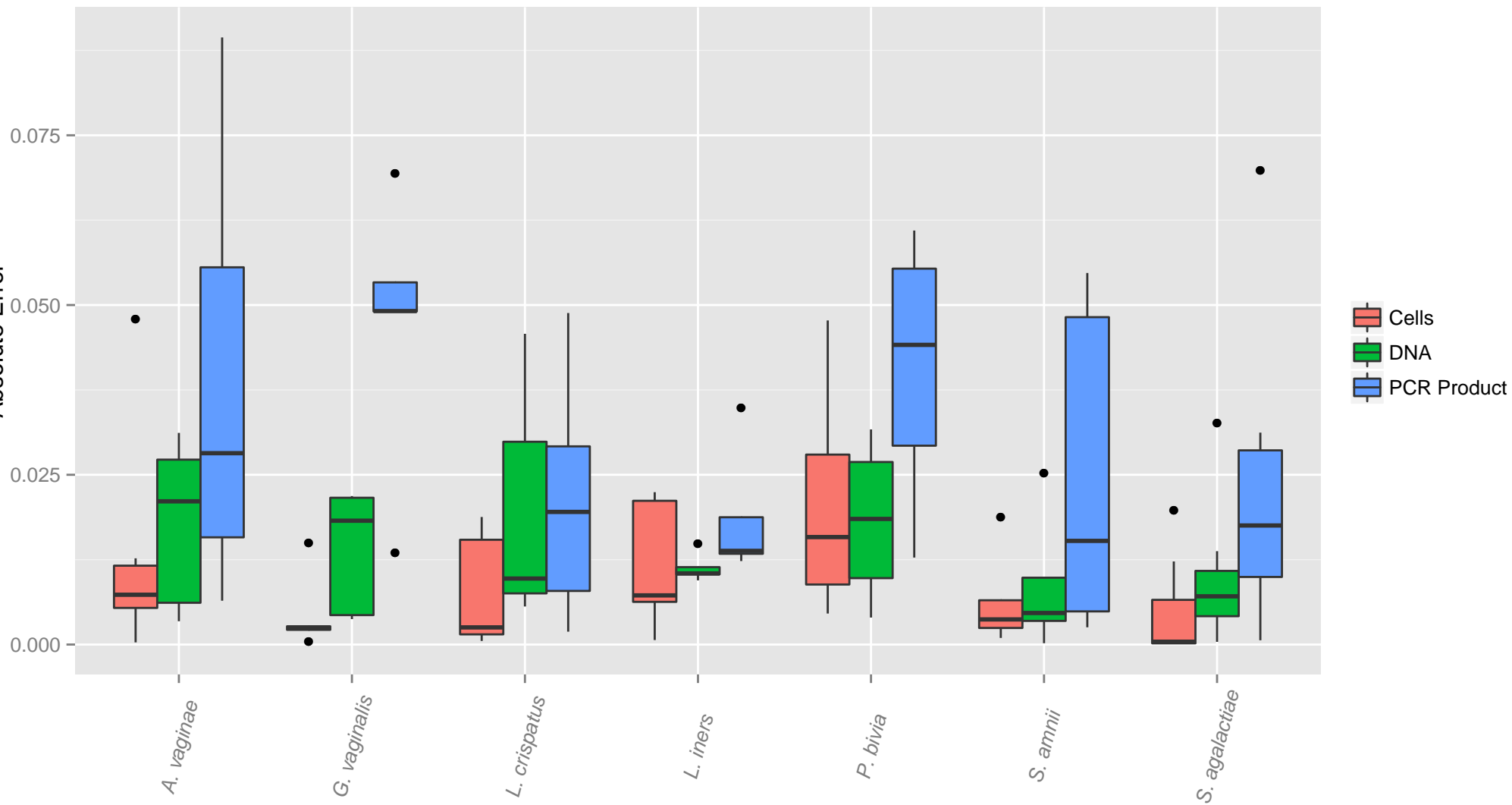

Supplement: Additional file 3 — Plot of technical variation in observed proportions of bacteria. Boxplot of the technical variation, measured as the absolute difference between observed proportions in replicate samples, for each bacterium and for experiments mixing equal proportions of cells, DNA, and PCR product. [file 12866_2015_351_MOESM3_ESM.pdf]

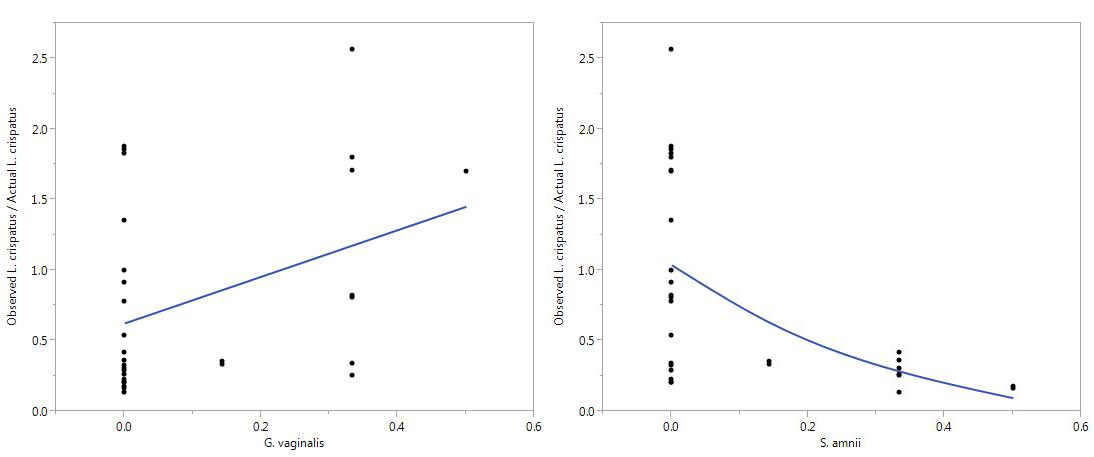

Supplement: Additional file 5 — Alternate visualization of Figure 3 with data. (left) The ratio of observed to actual L. crispatus versus the actual G. vaginalis for the samples, and the expected values based on mixture effect models. (right) The ratio of observed to actual L. crispatus versus the actual S. amnii for the samples, and the expected values based on mixture effect models. [file 12866_2015_351_MOESM5_ESM.jpeg]
