# Supplementary material for: The truth about metagenomics: quantifying and counteracting bias in 16S rRNA studies
Source: BMC Microbiol. 2015 Mar 21;15:66. doi: 10.1186/s12866-015-0351-6 (PMC4433096; doi:10.1186/s12866-015-0351-6)
Supplement: Additional file 7 — Code, comments, and output of analysis in R. R code, comments, figures, and results for analyzing the data from the experiments mixing equal amounts of cells, DNA, and PCR product. Created using R Markdown and knitr. The code demonstrates the use of Additional file 8 through Additional file 14. [file 12866_2015_351_MOESM7_ESM.pdf]

Code to Accompany “The Truth about Metagenomics: Quantifying and Counteracting Bias in 16S rRNA Studies”

Raw read data are available at SRA under BioProject PRJNA267701.

We use the STIRRUPS pipeline described in the paper by Fettweis et al.

The assignment of each read is in AdditionalFile15.txt.bz2. These results are rolled up based on the STIRRUPS and RDP classification to produce AdditionalFile8.txt.

The Python script AdditionalFile9.py takes as input the file AdditionalFile8.txt and produces a table of above-threshold counts AdditionalFile10.csv and below-threshold counts AdditionalFile11.csv.

AdditionalFile2.csv contains the design. This file contains the design and accession numbers and may be useful as a key.

Place Additional Files 2, 8-11 in a single directory, and the following R code produces the results below.

```
system("python AdditionalFile9.py")
```

Load libraries.

```
library(reshape2)
library(ggplot2)
library(plotrix)
library(bootstrap)
library(randomForest)
set.seed(12345)
```

Define column names.

```
organismsDesign <- c("Gvaginalis", "Avaginae", "Lcrispatus", "Liners", "Pbivia", "Samnii",
                     "GroupBStrep")
organismsResults <- c("Gardnerella.vaginalis", "Atopobium.vaginae",
                      "Lactobacillus.crispatus_cluster", "Lactobacillus.iners",
                      "Prevotella.bivia", "Sneathia.amnii", "Streptococcus.agalactiae")
organismsForPlots <- c("G. vaginalis", "A. vaginae", "L. crispatus", "L. iners",
                       "P. bivia", "S. amnii", "S. agalactiae")
```

Genome size and copy numbers obtained from NCBI.

```
genomeSize <- c(1.65, 1.43, 2.04, 1.3, 2.47, 1.34, 2.2)
copyNumbers <- c(2,1,4,1,1,1,7)
```

Read above-threshold counts data. Remove last (blank) column.

```
countdata <- read.table("AdditionalFile10.csv", sep=",", header=TRUE, row.names=1,
                        colClasses=c("character", rep("numeric", 46)))
countdata <- countdata[,-c(ncol(countdata))]
atOrganisms <- colnames(countdata)
```

Summarize the number of counts per sample.

```
atcounts <- apply(countdata,1,sum)
summary(atcounts)
```

```
##    Min. 1st Qu.  Median    Mean 3rd Qu.    Max.
##    4792   12200   14480   15290   17730   51580
```

Calculate the total number above-threshold reads.

```
sum(atcounts)
```

```
## [1] 3668922
```

Read the below-threshold counts data.

```
btdata <- read.table("AdditionalFile11.csv", sep=",", header=TRUE, row.names=1)
btdata <- btdata[,-c(ncol(btdata))]
btOrganisms <- sub("BT","", colnames(btdata))
```

Parse the sample IDs to get the plate and barcode numbers.

```
m <-  regexec("([1-6])_([0-9]+)" , rownames(countdata))
matchlist <- regmatches(rownames(countdata), m)
matchlistmatrix <- matrix(unlist(matchlist), ncol=3, byrow=TRUE)
Plate <- as.numeric(matchlistmatrix[,2])
Barcode <- as.numeric(matchlistmatrix[,3])
```

Join above-threshold counts with plate and barcode numbers.

```
atdata <- data.frame(countdata, Plate, Barcode)
atdata <- atdata[order(Plate,Barcode),]
```

Join below-threshold counts with plate and barcode numbers.

```
btdata <- data.frame(btdata, Plate, Barcode)
btdata <- btdata[order(Plate, Barcode),]
```

Read in the design from AdditionalFile2.csv and merge with the above-threshold counts data.

```
design <- read.table("AdditionalFile2.csv", sep=",", header=TRUE, row.names=1,
                     colClasses = c(rep("character",2), rep("numeric",9)) )

alldata <- merge(design, atdata, by=c("Plate", "Barcode"), all=TRUE)
alldata[,4:ncol(alldata)] <- sapply(alldata[,4:ncol(alldata)], as.numeric)
```

Label each sample according to the experiment. Experiment 1 mixed equal numbers of cells, Experiment 2 mixed equal DNA, Experiment 3 mixed equal PCR product.

```
experiment <- numeric(nrow(alldata))
experiment[(alldata$Plate == 1) | (alldata$Plate == 2)] <- 1
experiment[(alldata$Plate == 3) | (alldata$Plate == 4)] <- 2
experiment[(alldata$Plate == 5) | (alldata$Plate == 6)] <- 3
experiment <- factor(experiment)
```

Get the number of above-threshold reads classified as belonging to taxa that were not in the study.

```
otherData <- alldata[,-match(c("Plate","Barcode","Experiment",
                               organismsDesign, organismsResults), names(alldata))]
otherCounts <- apply(as.matrix(otherData),1,sum)
sum(otherCounts)
```

```
## [1] 733
```

Get the number of below-threshold reads classified as belonging to taxa not in the study.

```
allbtdata <- merge(btdata, atdata, by=c("Plate", "Barcode"), all=TRUE)
allbtdata <- sapply(allbtdata, as.numeric)

btexp1 <- allbtdata[(allbtdata[,"Plate"] == 1) | (allbtdata[,"Plate"] == 2),]
btexp2 <- allbtdata[(allbtdata[,"Plate"] == 3) | (allbtdata[,"Plate"] == 4),]
btexp3 <- allbtdata[(allbtdata[,"Plate"] == 5) | (allbtdata[,"Plate"] == 6),]

totalcounts1 <- apply(btexp1,1,sum)
totalcounts2 <- apply(btexp2,1,sum)
totalcounts3 <- apply(btexp3,1,sum)

btCounts1 <- btexp1[,-match(names(atdata), colnames(btexp1))]
btCounts2 <- btexp2[,-match(names(atdata), colnames(btexp2))]
btCounts3 <- btexp3[,-match(names(atdata), colnames(btexp3))]

btResultsOrganisms <- paste(organismsResults, "BT", sep="")

btNotResultsCounts1 <- sum(btCounts1[,-match(btResultsOrganisms, colnames(btCounts1))])
btNotResultsCounts2 <- sum(btCounts2[,-match(btResultsOrganisms, colnames(btCounts2))])
btNotResultsCounts3 <- sum(btCounts3[,-match(btResultsOrganisms, colnames(btCounts3))])

btNotResultsCounts1 + btNotResultsCounts2 + btNotResultsCounts3
```

```
## [1] 2279
```

Get total number of reads (above- and below-threshold).

```
sum(totalcounts1)+sum(totalcounts2)+sum(totalcounts3)
```

```
## [1] 3927760
```

Normalize the above-threshold data to proportions.

```r
dataNorm <- data.frame(alldata[,c(organismsDesign, organismsResults)], otherCounts)
Normcounts <- apply(dataNorm[,c(organismsResults, "otherCounts")],1,sum)
classcounts <- Normcounts
dataNorm[,c(organismsResults, "otherCounts")] <-
  dataNorm[,c(organismsResults, "otherCounts")]/Normcounts
```

Adjust counts data for copy number and genome size.

```r
dataNorm[experiment == 1,organismsResults] <-
  t(t(dataNorm[experiment == 1,organismsResults])/copyNumbers)
dataNorm[experiment == 2,organismsResults] <-
  t(t(dataNorm[experiment == 2,organismsResults])*(genomeSize/copyNumbers))
```

Re-normalize to proportions.

```r
Normcounts <- apply(dataNorm[,c(organismsResults, "otherCounts")],1,sum)
dataNorm[,c(organismsResults, "otherCounts")] <-
  dataNorm[,c(organismsResults, "otherCounts")]/Normcounts
```

Summarize the proportion of above-threshold reads classified as belonging to taxa not in the study for each sample.

```r
summary(dataNorm$otherCounts)
```

```
##      Min.   1st Qu.    Median      Mean   3rd Qu.      Max.
## 0.000e+00 0.000e+00 4.529e-05 3.559e-04 1.977e-04 2.723e-02
```

Make a data frame for each experiment.

```r
classcounts1 <- classcounts[experiment == 1]
classcounts2 <- classcounts[experiment == 2]
classcounts3 <- classcounts[experiment == 3]

exp1Norm <- dataNorm[experiment == 1,]
exp2Norm <- dataNorm[experiment == 2,]
exp3Norm <- dataNorm[experiment == 3,]
```

Re-order the rows according to the design.

```r
classcounts1 <- classcounts1[with(exp1Norm, order(Gvaginalis, Avaginae, Lcrispatus, Liners,
                                                  Pbivia, Samnii, GroupBStrep))]
classcounts2 <- classcounts2[with(exp2Norm, order(Gvaginalis, Avaginae, Lcrispatus, Liners,
                                                  Pbivia, Samnii, GroupBStrep))]
classcounts3 <- classcounts3[with(exp3Norm, order(Gvaginalis, Avaginae, Lcrispatus, Liners,
                                                  Pbivia, Samnii, GroupBStrep))]

exp1Norm <- exp1Norm[with(exp1Norm, order(Gvaginalis, Avaginae, Lcrispatus, Liners,
                                          Pbivia, Samnii, GroupBStrep)),]
exp2Norm <- exp2Norm[with(exp2Norm, order(Gvaginalis, Avaginae, Lcrispatus, Liners,
                                          Pbivia, Samnii, GroupBStrep)),]
exp3Norm <- exp3Norm[with(exp3Norm, order(Gvaginalis, Avaginae, Lcrispatus, Liners,
                                          Pbivia, Samnii, GroupBStrep)),]
```

Plot of centroid for each experiment.

```
centroidPlotData <- rbind(c(rep(1/7,7),0),
  exp1Norm[apply(exp1Norm[,organismsDesign] > 0,1,sum) == 7, c(organismsResults, "otherCounts")],
  exp2Norm[apply(exp2Norm[,organismsDesign] > 0,1,sum) == 7,c(organismsResults, "otherCounts")],
  exp3Norm[apply(exp3Norm[,organismsDesign] > 0,1,sum) == 7,c(organismsResults, "otherCounts")])

mycolors <- c("red", "brown", "yellow", "lightblue", "green", "purple", "orange", "pink")
```

The observed proportions after mixing equal numbers of cells.

```
pie3D(as.numeric(100*centroidPlotData[2,1:7]), explode=0.1, col=mycolors[1:7],
      labels=organismsForPlots)
```

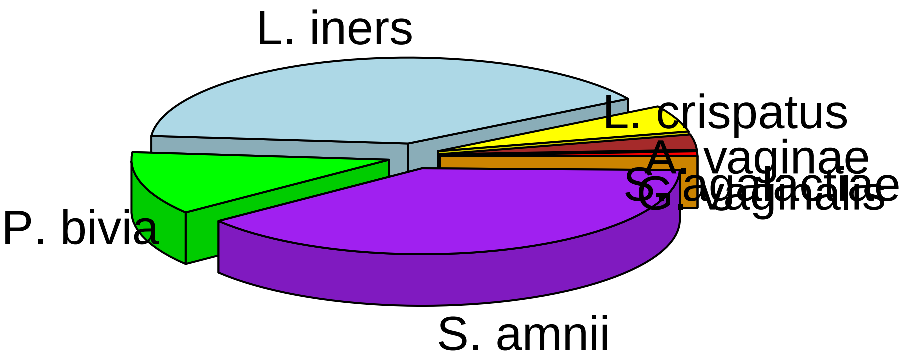

The observed proportions after mixing equal amounts of DNA.

```
pie3D(as.numeric(100*centroidPlotData[4,1:7]), labels=organismsForPlots,
      explode=0.1, col=mycolors)
```

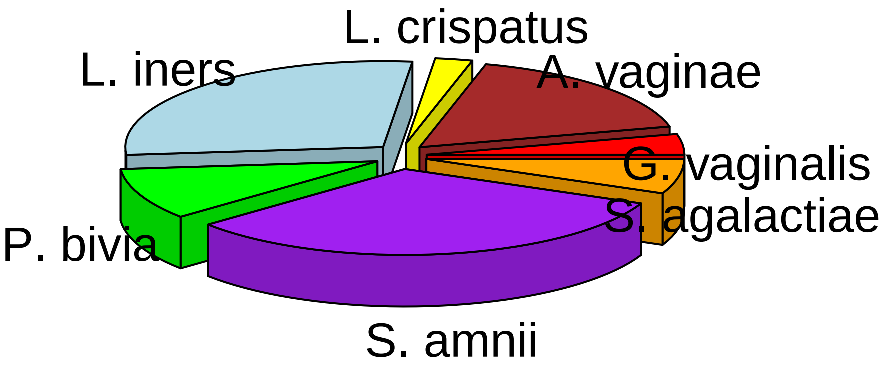

The observed proportions after mixing equal amounts of PCR product.

```
pie3D(as.numeric(100*centroidPlotData[6,1:7]), labels=organismsForPlots,
      explode=0.1, col=mycolors)
```

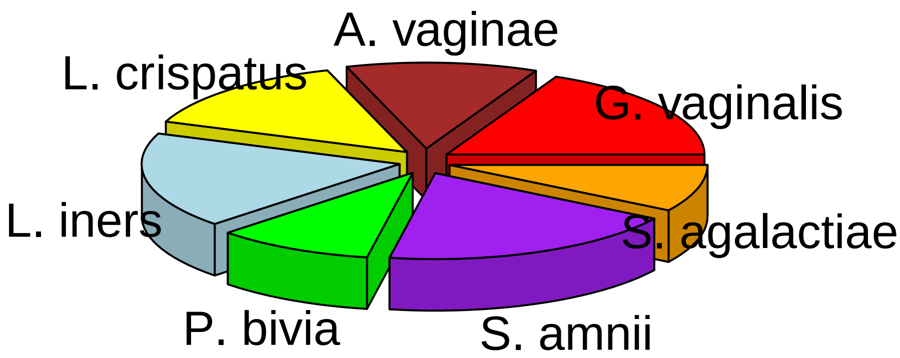

Plot of actual and observed proportions for each experiment for the samples mixing equal amounts of *L. crispatus* and *S. agalactiae*.

```
truth <- rep(0,length(organismsDesign)+1)
truth[c(organismsDesign,"other") == "GroupBStrep"] <- 0.5
truth[c(organismsDesign,"other") == "Lcrispatus"] <- 0.5
crispStrepPlotData <- rbind(truth, dataNorm[dataNorm$Lcrispatus == 0.5
                  & dataNorm$GroupBStrep == 0.5,c(organismsResults, "otherCounts")])
crispStrepPlotData <- rbind(truth, 0.5*(crispStrepPlotData[2,] + crispStrepPlotData[3,]),
                             0.5*(crispStrepPlotData[4,] + crispStrepPlotData[5,]),
                             0.5*(crispStrepPlotData[6,] + crispStrepPlotData[7,]))
mysalabels <- c("Actual", "Exp. 1", "Exp. 2", "Exp. 3")
par(xpd=T, mar=c(5,4,4,10)+1.0)
barplot(100*t(crispStrepPlotData),ylim=c(0,100),col=mycolors, axisnames=TRUE,
        names.arg=mysalabels, ylab="Percentage of Reads (%)")
legend(x=5,y=100, c(organismsForPlots, "Other"), fill=mycolors)
```

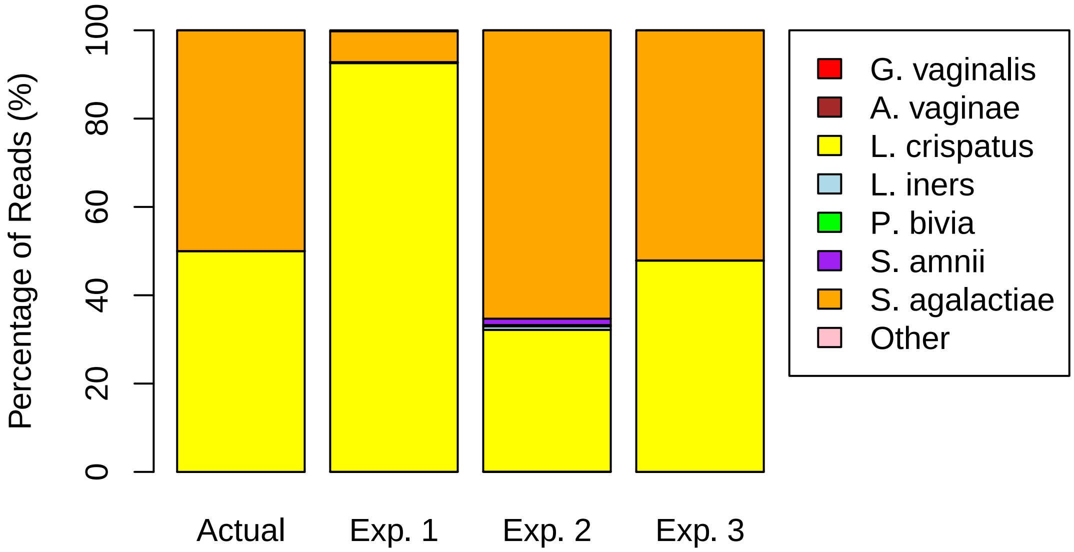

Box plot of bias at each step.

```
dnaExtDiff <- vector("list", 7)
pcrAmpDiff <- vector("list", 7)
seqClaDiff <- vector("list", 7)
totExpDiff <- vector("list", 7)
```

```
names(dnaExtDiff) <- organismsDesign
names(pcrAmpDiff) <- organismsDesign
names(seqClaDiff) <- organismsDesign
names(totExpDiff) <- organismsDesign

for (i in 1:length(organismsDesign)) {
 dnaExtDiff[[i]] <- 100*exp1Norm[exp1Norm[,i] != 0, i+7] - 100*exp2Norm[exp2Norm[,i] !=0, i+7]
 pcrAmpDiff[[i]] <- 100*exp2Norm[exp2Norm[,i] != 0, i+7] - 100*exp3Norm[exp3Norm[,i] !=0, i+7]
 seqClaDiff[[i]] <- 100*exp3Norm[exp3Norm[,i] != 0, i+7] - 100*exp3Norm[exp3Norm[,i] !=0, i]
 totExpDiff[[i]] <- 100*exp1Norm[exp1Norm[,i] != 0, i+7] - 100*exp1Norm[exp1Norm[,i] !=0, i]
}

expPlotData <- list(dnaExtDiff, pcrAmpDiff, seqClaDiff, totExpDiff)
names(expPlotData) <- c("DNA Extraction", "PCR Amplification",
                        "Sequencing\nand Classification","Total")

expBoxPlot <- ggplot(melt(expPlotData), aes(x=L2, y=value)) + geom_boxplot(aes(fill=L1))
expBoxPlot <- expBoxPlot +
       scale_x_discrete(breaks=c(organismsDesign), labels=organismsForPlots) +
       theme(axis.text.x = element_text(angle=75, vjust=0.5, face="italic")) +
       ylab("Bias (% Difference)") +
       xlab("") +scale_fill_discrete(name="")
expBoxPlot
```

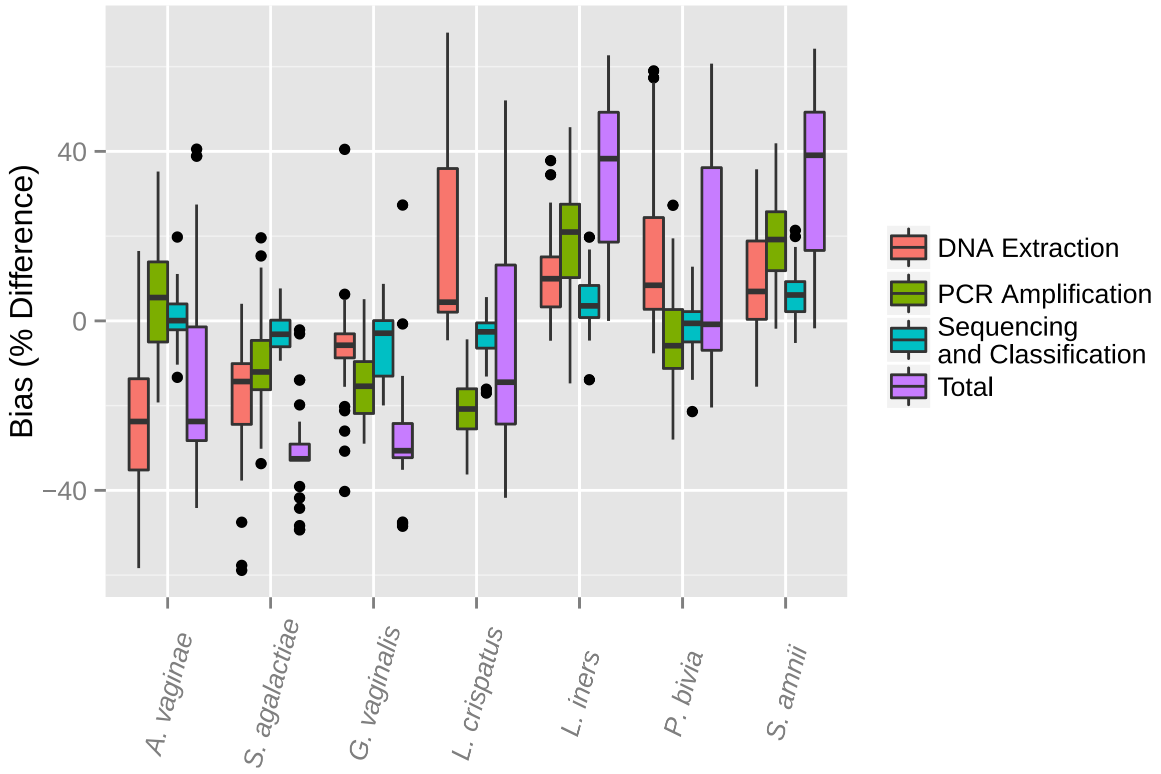

Write data for JMP to build mixture models.

```
write(t(cbind(exp1Norm,classcounts1)),file="AdditionalFile12.txt", ncolumns=ncol(exp1Norm)+1)
write(t(cbind(exp2Norm,classcounts2)),file="AdditionalFile13.txt", ncolumns=ncol(exp2Norm)+1)
write(t(cbind(exp3Norm,classcounts3)),file="AdditionalFile14.txt", ncolumns=ncol(exp3Norm)+1)
```

Test if bias is significantly different from zero with bootstrap test.

```
paired <- function(x, data1)
{
 t <- (mean(data1[x])-mean(data1))/(sd(data1[x])/sqrt(length(data1[x])))
}

num.boot=10000
bootpaired <- function(data2)
{
 tdata <- mean(data2)/(sd(data2)/sqrt(length(data2)))
 mylist <- bootstrap(1:length(data2), num.boot, paired, data2)
 pval <- sum(mylist$thetastar > abs(tdata))/num.boot
}

dnaExt.pval <- cbind(melt(lapply(dnaExtDiff,mean)),melt(lapply(dnaExtDiff,bootpaired)))
pcrAmp.pval <- cbind(melt(lapply(pcrAmpDiff,mean)),melt(lapply(pcrAmpDiff,bootpaired)))
seqCla.pval <- cbind(melt(lapply(seqClaDiff,mean)),melt(lapply(seqClaDiff,bootpaired)))
```

Apply a Bonferoni correction.

```
dnaExt.pval.adj <- dnaExt.pval[,3]*7
pcrAmp.pval.adj <- pcrAmp.pval[,3]*7
seqCla.pval.adj <- seqCla.pval[,3]*7

names(dnaExt.pval.adj) <- organismsForPlots
names(pcrAmp.pval.adj) <- organismsForPlots
names(seqCla.pval.adj) <- organismsForPlots
```

Test if bias due to DNA extraction is significantly different from zero.

```
dnaExt.pval.adj
```

```
##  G. vaginalis    A. vaginae  L. crispatus      L. iners      P. bivia
##        0.0154        0.0000        0.0000        0.0000        0.0000
##      S. amnii S. agalactiae
##        0.0042        0.0000
```

Test if bias due to PCR amplification is significantly different from zero.

```
pcrAmp.pval.adj
```

```
##  G. vaginalis    A. vaginae  L. crispatus      L. iners      P. bivia
##        0.0000        0.0791        0.0000        0.0000        1.1452
##      S. amnii S. agalactiae
##        0.0000        0.0000
```

Test if bias due to sequencing and classification is significantly different from zero.

```
seqCla.pval.adj
```

```
##  G. vaginalis    A. vaginae  L. crispatus      L. iners      P. bivia
##        0.0112        1.6058        0.0189        0.0098        0.8750
##      S. amnii S. agalactiae
##        0.0000        0.0238
```

Calculate and plot technical variation by calculating differences between replicate samples.

```
mspe <- function(x) {
 if (length(x) == 1) {
   NA
 } else {
   sum((x - mean(x))^2/length(x))
 }
}

techError <- function(x) {
 if (length(x) == 1) {
   NA } else {
   abs((x - mean(x)))
 }
}

techVarExp1 <- aggregate(exp1Norm[,organismsResults],by=exp1Norm[,organismsDesign],
                         FUN=techError)
techVarExp2 <- aggregate(exp2Norm[,organismsResults],by=exp2Norm[,organismsDesign],
                         FUN=techError)
techVarExp3 <- aggregate(exp3Norm[,organismsResults],by=exp3Norm[,organismsDesign],
                         FUN=techError)

techVarExp1Res <- techVarExp1[,organismsResults]
techVarExp2Res <- techVarExp2[,organismsResults]
techVarExp3Res <- techVarExp3[,organismsResults]

techVarExp1Des <- techVarExp1[,organismsDesign]
techVarExp2Des <- techVarExp2[,organismsDesign]
techVarExp3Des <- techVarExp3[,organismsDesign]

techVarExp1Res[techVarExp1Des == 0] <- NA
techVarExp2Res[techVarExp2Des == 0] <- NA
techVarExp3Res[techVarExp3Des == 0] <- NA

techVarExp1Res <- apply(techVarExp1Res,2,unlist)
techVarExp2Res <- apply(techVarExp2Res,2,unlist)
techVarExp3Res <- apply(techVarExp3Res,2,unlist)

techVarExp1Res <- sapply(techVarExp1Res,na.omit)
techVarExp2Res <- sapply(techVarExp2Res,na.omit)
techVarExp3Res <- sapply(techVarExp3Res,na.omit)

techPlotData <- list(techVarExp1Res, techVarExp2Res, techVarExp3Res)
names(techPlotData) <- c("Cells", "DNA", "PCR Product")
```

```r
techBoxPlot <- ggplot(melt(techPlotData), aes(x=L2,y=value)) + geom_boxplot(aes(fill=L1))
techBoxPlot <- techBoxPlot +
        scale_x_discrete(breaks=c(organismsResults), labels=organismsForPlots) +
        theme(axis.text.x = element_text(angle=75, vjust=0.5, face="italic")) +
        ylab("Absolute Error") +
        xlab("") +
        scale_fill_discrete(name="")
techBoxPlot
```

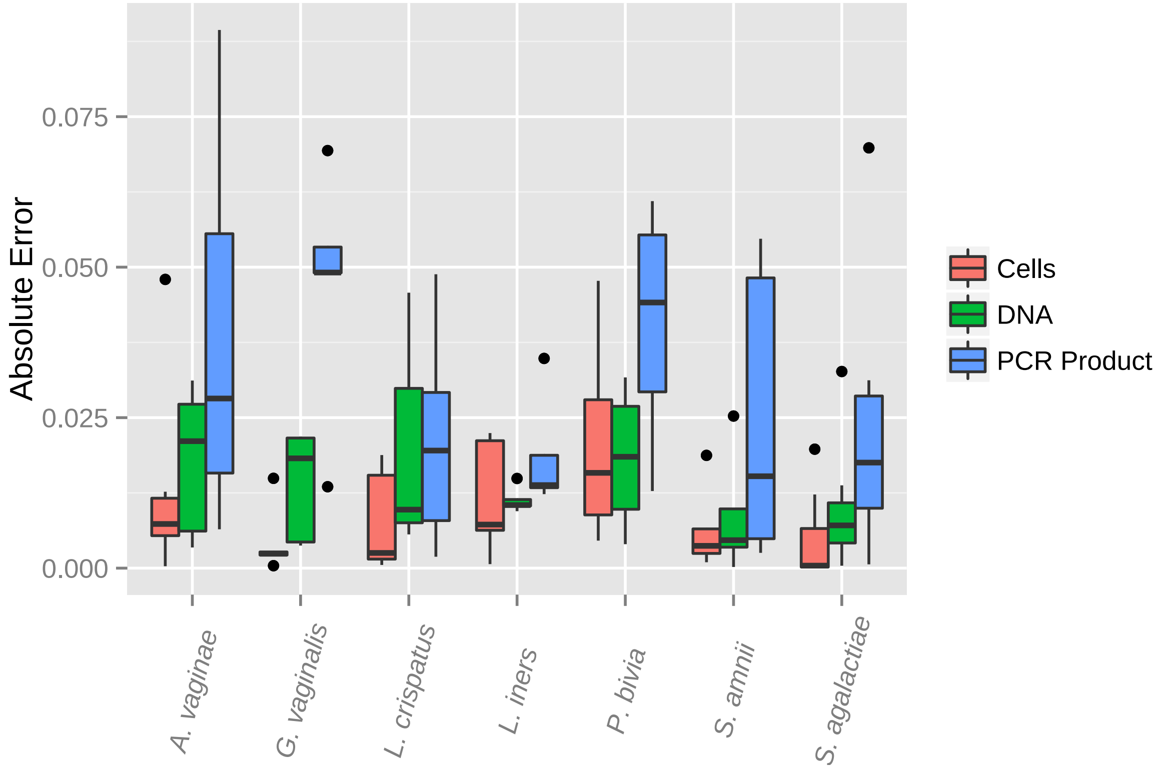

Plot observed proportions for samples from a human subject.

```r
collectionDays <- c(0,195,213,155)
samplepH <- c("5.0","5.0","4.4","5.5")
dxclinician <- c("Bacterial Vaginosis", "Annual Exam", "Yeast Infection", "Annual Exam")
clinicalData <- matrix(
  c(30.21596,61.97393,1.40667,52.56851,0.03755869,24.97517,0.02689618,10.89964,
    0,0.003103662,0.008068854,0.1982554,43.94366,8.361266,97.21356,30.64147,0,
    0.06517691,0,0.1806327,0.07511737,0.04034761,0, 0.176227,0,0,0,0,25.7277,
    4.581006,1.344809,5.335272)/100,ncol=8)
colnames(clinicalData) <- c(organismsResults, "otherCol")
clinicalPlotData <- 100*clinicalData

mylabels <- paste(collectionDays, samplepH, dxclinician, sep="\n")
par(xpd=T, mar=c(5,4,4,10)+1.0)
barplot(t(clinicalPlotData), ylim=c(0,100), las=1, col=mycolors, axisnames=TRUE,
        names.arg=mylabels, ylab="Percentage of Reads (%)", cex.names=0.6, mgp=c(4,3,0))
legend(x=5,y=100, c(organismsForPlots, "Other"), fill=mycolors)
```

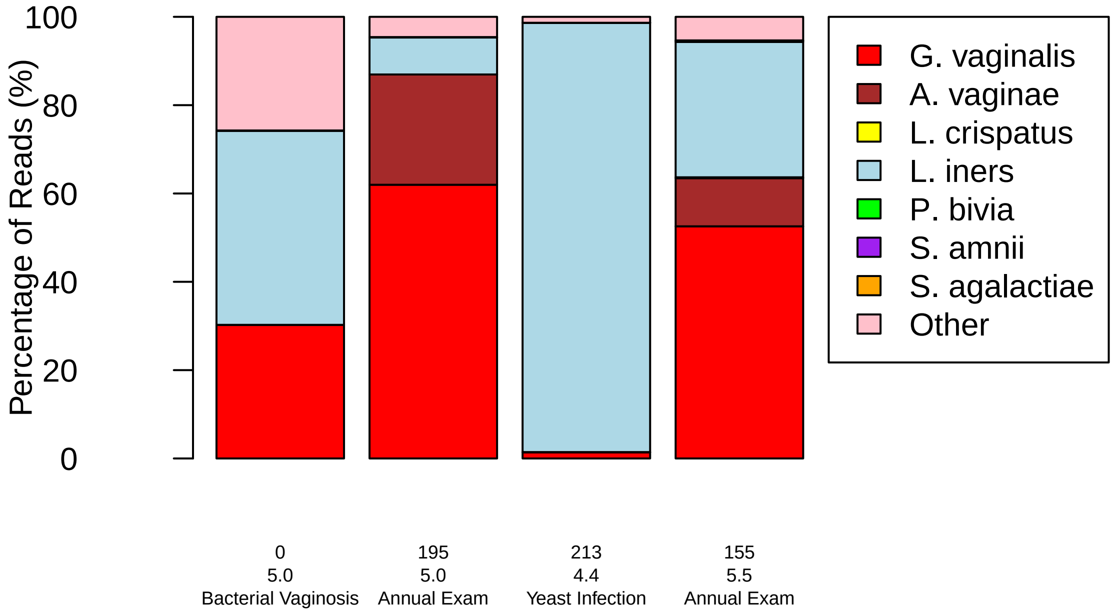

Prepare data for predictive models by adjusting for copy number.

```
clinicalPredData <- as.matrix(clinicalData[,1:7])
clinicalPredData <- t(t(clinicalPredData)/copyNumbers)
clinicalPredData <- clinicalPredData/apply(clinicalPredData,1,sum)
clinicalPredData <- data.frame(clinicalPredData)
```

Use random forests to build models predicting the scaling factor to apply to observed proportions to get the actual proportions in the mock communities.

```
train <- exp1Norm[,8:14]
resp <- exp1Norm[,1:7]

scaledf <- resp/train
scaledf[is.na(scaledf)]<-0

train1 <- train
scaledf1 <- scaledf[,1]
train2 <- train
scaledf2 <- scaledf[,2]
train3 <- train
scaledf3 <- scaledf[,3]
train4 <- train
scaledf4 <- scaledf[,4]
train5 <- train
scaledf5 <- scaledf[,5]
train6 <- train
scaledf6 <- scaledf[,6]
train7 <- train
scaledf7 <- scaledf[,7]

myrf1 <- randomForest(x=train1,y=scaledf1,ntree=1000)
myrf2 <- randomForest(x=train2,y=scaledf2,ntree=1000)
myrf3 <- randomForest(x=train3,y=scaledf3,ntree=1000)
```

```
myrf4 <- randomForest(x=train4,y=scaledf4,ntree=1000)
myrf5 <- randomForest(x=train5,y=scaledf5,ntree=1000)
myrf6 <- randomForest(x=train6,y=scaledf6,ntree=1000)
myrf7 <- randomForest(x=train7,y=scaledf7,ntree=1000)
```

Apply the models to the clinical samples.

```
mynames<-names(clinicalPredData)
names(clinicalPredData) <- names(train)

mypreds1 <- predict(myrf1,clinicalPredData)
mypreds2 <- predict(myrf2,clinicalPredData)
mypreds3 <- predict(myrf3,clinicalPredData)
mypreds4 <- predict(myrf4,clinicalPredData)
mypreds5 <- predict(myrf5,clinicalPredData)
mypreds6 <- predict(myrf6,clinicalPredData)
mypreds7 <- predict(myrf7,clinicalPredData)
preds1 <- data.frame(V1pred= mypreds1, V2pred= mypreds2,V3pred = mypreds3,
                     V4pred = mypreds4,V5pred = mypreds5, V6pred=mypreds6,V7pred=mypreds7)
preds1 <- preds1*(clinicalPredData*(clinicalPredData>.001))
clinicalPredicted <- t(apply(preds1,1,function(x){x<-x/sum(x)}))
round(clinicalPredicted,digits=4)
```

```
##   V1pred V2pred V3pred V4pred V5pred V6pred V7pred
## 1 0.9302 0.0000      0 0.0698      0      0      0
## 2 0.7999 0.1998      0 0.0003      0      0      0
## 3 0.2005 0.0000      0 0.7995      0      0      0
## 4 0.8723 0.1049      0 0.0227      0      0      0
```

Plot predicted clinical values.

```
clinicalPredicted <- 100*clinicalPredicted*(1-clinicalData[,8])
clinicalPredicted <- data.frame(clinicalPredicted, 100*clinicalData[,8])

par(xpd=T, mar=c(5,4,4,10)+1.0)
barplot(t(clinicalPredicted), ylim=c(0,100), las=1, col=mycolors,
        axisnames=TRUE, ylab="Percentage of Reads (%)", names.arg=mylabels,
        mgp=c(4,3,0), cex.names=0.6)
legend(x=5,y=100, c(organismsForPlots, "Other"), fill=mycolors)
```

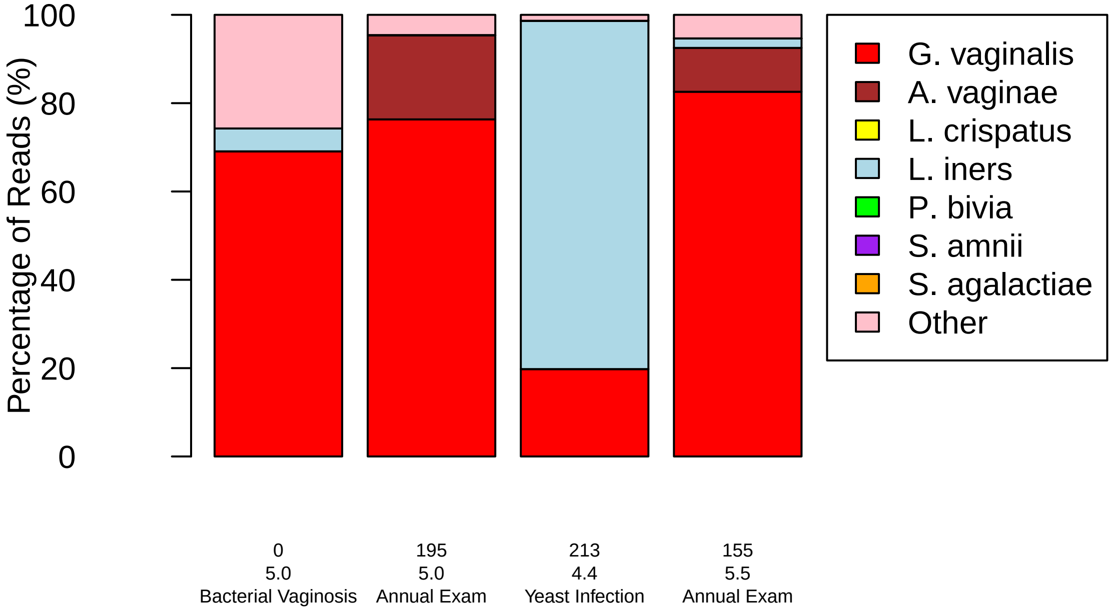
Percentage of Reads (%)
100
80
60
40
20
0
G. vaginalis
A. vaginae
L. crispatus
L. iners
P. bivia
S. amnii
S. agalactiae
Other
0
5.0
Bacterial Vaginosis
195
5.0
Annual Exam
213
4.4
Yeast Infection
155
5.5
Annual Exam
